# Integrating the prevention of mother-to-child transmission of HIV into primary healthcare services after AIDS denialism in South Africa: perspectives of experts and health care workers - a qualitative study

**DOI:** 10.1186/s12913-020-05381-5

**Published:** 2020-06-26

**Authors:** Jean Claude Mutabazi, Corie Gray, Lorrein Muhwava, Helen Trottier, Lisa Jayne Ware, Shane Norris, Katherine Murphy, Naomi Levitt, Christina Zarowsky

**Affiliations:** 1 Département de médecine sociale et préventive, École de Santé Publique, Université de Montréal, Pavillon 7101, Avenue du Parc, Montréal, QC H3N 1X7 Canada; 2 Centre de recherche en santé publique (CReSP), Université de Montréal et CIUSSS du Centre-Sud-de-l’Île-de-Montréal, Montréal, QC, H3L 1M3 Canada; 3 Centre de Recherche du Centre Hospitalier Universitaire Sainte Justine, Montréal, QC H3T 1C5, Canada; 4 Collaboration for Evidence, Research and Impact in Public Health, School of Public Health, Curtin University, Kent Street, Bentley, Perth, Western Australia 6102, Australia; 5 Division of Endocrinology, Department of Medicine, Faculty of Health Science, University of Cape Town, Chronic Disease Initiative for Africa, J Floor, Old Main Building, Groote Schuur Hospital, Observatory 7925, Cape Town, Western Cape South Africa; 6 Developmental Pathways for Health Research Unit, Department of Paediatrics, School of Clinical Medicine, University of the Witwatersrand, 26 Chris Hani Road, Chris Hani Baragwanath Academic Hospital, Soweto, Johannesburg, South Africa; 7 School of Public Health, University of the Western Cape, Robert Sobukwe Rd, Bellville 7535, Cape Town, Western Cape, South Africa

**Keywords:** Integration, Health systems, Primary health care, Prevention of mother-to-child transmission of HIV, PMTCT programme, PMTCT service, HIV prevention, South Africa

## Abstract

**Background:**

Integrating Prevention of Mother-to-Child Transmission (PMTCT) programmes into routine health services under complex socio-political and health system conditions is a priority and a challenge. The successful rollout of PMTCT in sub-Saharan Africa has decreased Human Immunodeficiency Virus (HIV), reduced child mortality and improved maternal health. In South Africa, PMTCT is now integrated into existing primary health care (PHC) services and this experience could serve as a relevant example for integrating other programmes into comprehensive primary care. This study explored the perspectives of both experts or key informants and frontline health workers (FHCWs) in South Africa on PMTCT integration into PHC in the context of post-AIDS denialism using a Complex Adaptive Systems framework.

**Methods:**

A total of 20 in-depth semi-structured interviews were conducted; 10 with experts including national and international health systems and HIV/PMTCT policy makers and researchers, and 10 FHCWs including clinic managers, nurses and midwives. All interviews were conducted in person, audio-recorded and transcribed. Three investigators collaborated in coding transcripts and used an iterative approach for thematic analysis.

**Results:**

Experts and FHCWs agreed on the importance of integrated PMTCT services. Experts reported a slow and partial integration of PMTCT programmes into PHC following its initial rollout as a stand-alone programme in the aftermath of the AIDS denialism period. Experts and FHCWs diverged on the challenges associated with integration of PMTCT. Experts highlighted bureaucracy, HIV stigma and discrimination and a shortage of training for staff as major barriers to PMTCT integration. In comparison, FHCWs emphasized high workloads, staff turnover and infrastructural issues (e.g., lack of rooms, small spaces) as their main challenges to integration. Both experts and FHCWs suggested that working with community health workers, particularly in the post-partum period, helped to address cases of loss to follow-up of women and their babies and to improve linkages to polymerase-chain reaction (PCR) testing and immunisation.

**Conclusions:**

Despite organised efforts in South Africa, experts and FHCWs reported multiple barriers for the full integration of PMTCT in PHC, especially postpartum. The results suggest opportunities to address operational challenges towards more integrated PMTCT and other health services in order to improve maternal and child health.

## Background

Health systems operate in conditions of increasing complexity, in which health care providers interact with patients and their families in the context of multiple diseases or health problems [1–3] as well as with managers and political decision makers concerned with successful implementation and scale-up [4, 5]. Integrating stand-alone interventions into comprehensive services at local facilities requires facing the challenges of limited health service accessibility, critical socio-economic conditions and the double burden of communicable and non-communicable diseases [6–8]. Integration of the prevention of mother-to-child transmission (PMTCT) of human immunodeficiency virus (HIV) programme in South Africa can serve as a case study of this adaptation under complex conditions – including the particularly challenging socio-political context of “AIDS denialism” in which PMTCT and other HIV-related interventions were developed and implemented in South Africa [9–11]. Efforts to integrate new interventions into routine primary care may be informed by reflecting on the technical and health system challenges of implementing the PMTCT cascade. In addition, the history of AIDS denialism and the challenges of initially launching PMTCT programmes in South Africa may shed light on adapting complex, costly and contested interventions into public health systems.

The transmission of HIV from mother to child can be reduced by more than 95% with effective interventions during pregnancy, labor, delivery and breastfeeding, known collectively as the PMTCT cascade [12, 13]. The PMTCT cascade encompasses services aiming to virtually eliminate mother-to-child transmission (eMTCT) of HIV, reduce child mortality and improve maternal health [14, 15]. Since first reported in the 1980s, the proportion of infections by MTCT has decreased from an estimated 10% of new HIV cases to less than 2% [16, 17]. The rollout of PMTCT has been particularly successful where the burden is greatest in low and middle income countries (LMICs) including in sub-Saharan Africa (SSA) and in South Africa [16, 17]. The acknowledgement of the impact of perinatal HIV on maternal and child health (MCH) [17, 18] saw PMTCT services receive significant attention from various actors at local, national and international levels. These stakeholders included organizations such as the United States President’s Emergency Plan for AIDS Relief (PEPFAR) and the Global Fund to fight against AIDS, tuberculosis (TB) and Malaria (GFATM), United States Agency for International Development (USAID), Centers for Disease Control (CDC) and international non-governmental organizations (NGOs), different governmental initiatives and local health systems [19]. This support allowed for the integration of PMTCT into existing health care services [20], with the ambition of achieving eMTCT [21, 22].

PMTCT has been integrated into existing services within PHC or community outreach programmes to prevent other diseases such as tuberculosis, diarrhea, depression and others [23, 24]. The impact of PMTCT on the improvement of MCH in SSA has been well documented, especially in improving service availability, accessibility and utilization [25, 26]. Unlike the situation for other health problems, this integration of PMTCT in PHC has provided the opportunity to improve preventive services and manage other common health issues within countries [24], rather than shifting scarce resources to vertical programmes [27]. Well-resourced PMTCT programmes have contributed to improving the quality and ensuring the sustainability of other services offered in the same clinics [28]. However, sustaining such results through national rather than donor funding and management, has increased pressure on some facilities that face shortages in terms of human resources and medical supplies, or that lack required knowledge and training to deliver full service packages [29].

These challenges of allocating scarce resources are political as well as technical. The South African experience highlights the interactions among politics, health care, and public health. Despite now having the world’s largest antiretroviral therapy (ART) programmes and well-established PMTCT rollout [30], South Africa’s initial response to HIV was stalled due to political interference in health policy during the time of Thabo Mbeki’s presidency from 1999 to 2008. Mbeki subscribed to the AIDS denialist view that retroviruses are harmless and that other influences, such as drug abuse, poverty, and antiretroviral medications cause AIDS [31]. Under his presidency, the government instituted policies denying antiretroviral drugs to AIDS patients and withdrew support from clinics that had started using azidothymidine (AZT) also known as zidovudine, to prevent mother-to-child transmission of HIV. In 1998, the Treatment Action Campaign (TAC) was founded, campaigning for measures to reduce new HIV infections. In 2001, TAC initiated legal proceedings against the government’s policy of limiting treatment for the purpose of PMTCT. The use of ARV/AZT only resumed in 2002 when the constitutional court ruled that the denial of treatment was an infringement of the fundamental right to healthcare (Section 27 & 28) [32]. In this time period an estimated 100,000 infants were believed to be infected [30, 33].

However, it was not until 2008 that the rapid implementation of PMTCT and ART services was prioritised [34]. In the period between the first Nevirapine regimen in 2002 and the national PMTCT plan in 2008 at least 330,000 preventable deaths were estimated to have occurred, with 35,000 babies born with HIV. The delay in the rollout of PMTCT caused inestimable financial and social loss, with HIV infection rates well above other SSA countries that had initiated PMTCT in the early 2000s [35, 36].

Since then, substantial achievements in PMTCT have been seen in South Africa. HIV testing coverage of pregnant women is now close to 100% and PMTCT services were provided in almost 98% of all health facilities by 2015 [37]. In 2011, MTCT decreased to 2.7%, with ART coverage reaching 87% in 2012 [34, 37]. These significant results were achieved following changes in National PMTCT guidelines to initiate treatment for all women living with HIV regardless of CD4 count. A timeline of South Africa’s PMTCT Guidelines is summarized in the adapted table below (Table 1) [30].

**Table 1:** Adapted timeline of national PMTCT guidelines, South Africa [30]

PMTCT Guidelines	Year
National PMTCT programme begins after Constitutional Court judgement against the Government: •Single dose nevirapine to the mother in labour, and to the infant within 72 h of birth.	2002
AZT to mother from 28 weeks gestation; single dose nevirapine to mother in labour and to infant within 72 h of birth and HAART for pregnant women.	2004
•AZT from 14 weeks gestation: single dose nevirapine plus tenofovir/3TC in labour; infant prophylaxis with nevirapine for 6 weeks if mother on HAART or formula feeding, or until the end of all breastfeeding if mother not eligible for HAART.	2010
•All pregnant women eligible for HAART, irrespective of CD4 count. Infant prophylaxis with nevirapine for 6 weeks. Women initiating HAART with CD4 < 350 and no other indication for HAART, to stop treatment after all breastfeeding has ceased.	2013
•All pregnant and breastfeeding women eligible for lifelong HAART.	2015

*AZT* zidovudine, *HAART* highly active antiretroviral therapy

There are, however, few reports of research findings regarding the process, successes and failures of PMTCT implementation [22, 38, 39]. Understanding the perspectives, experiences or practices of different health system and PMTCT specialists, alongside frontline health workers who have been involved in daily PMTCT activities at PHC level, could facilitate the further integration of PMTCT services and inform efforts to integrate other vertical programmes into PHC. The objective of this study was to explore these perspectives in the context of post-AIDS denialism in South Africa and document important considerations for the integration of other comprehensive services in maternal and child health care.

## Methods

### Study framework

For this study, an adapted Complex Adaptive Systems (CAS) framework to describe features explaining key components of integration of PMTCT [40], was used. There are four nested levels that can be considered for health care system changes and improvement [41, 42]. These include: individual patients; care teams (e.g., family members, nurses, doctors, pharmacists); organisation (e.g., clinics, hospitals, infrastructure, quality assurance); and the larger system or environment (e.g., public and private regulators, insurance companies, research funders, legislative and policy frameworks) [41, 42]. Many local, national and international actors, including individual or country initiatives and bilateral or multilateral organizations, also exist in PMTCT programmes.

**Fig. 1:**
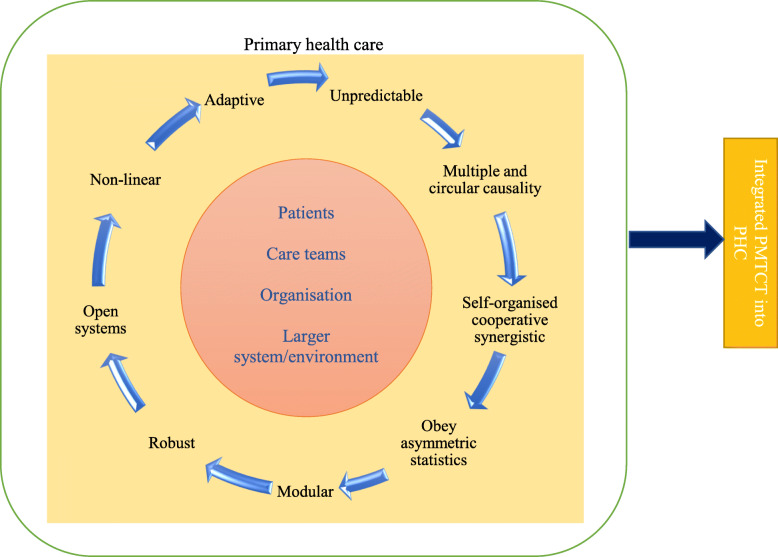
Adapted study framework towards integrated PMTCT into PHC [40, 43–46]

CAS has been proposed as a theory to both understand, and work towards, integrated health care [40, 43–46]. Health systems are perceived as “complex” because they imply a wide variety of components, “adaptive” due to the ability to adjust or change over time and “system” because of its group of connections or interdependence [46]. Though there is no true consensus on the set of characteristics that define CAS [47], Cilliers, P [48]. proposed a list of important CAS characteristics, later summarised by Maguire, S. et al. [49]. These characteristics can be applied to the South African health system and to PMTCT intervention and include: unpredictable, multiple with circular causality, self-organised cooperative synergistic, obey asymmetric statistics, modular, robust, open systems, non-linear and adaptive (these framework features are described in the supplemental material 1). Considering the complexity of the process and environmental/contextual elements, the following framework to understand the integration of PMTCT into PHC was proposed for this study (Fig. 1).

#### Study design and sample

A descriptive qualitative study to explore different perspectives and experiences regarding PMTCT integration in the context of AIDS denialism was conducted. Two groups of informants were interviewed. The first were experts or key informants including health systems and HIV/PMTCT policy makers, researchers and activists at international, national and provincial levels in South Africa. The second were FHCWs and these were drawn from PHC facilities in Cape Town, Western Cape Province, in order to inform an ongoing intervention study in that province on the feasibility of integrating post-partum care and diabetes prevention activities for women with gestational diabetes mellitus into routine practice in public sector well-baby clinics. The domains of the consolidated criteria for reporting qualitative research (COREQ) [50] guided the development of this research and reporting of the findings (Additional file 1).

Experts were purposively selected and their Recruitment occurred between June 2016 and October 2017. Eligibility criteria included experience in design or implementation of PMTCT in South Africa and other regional countries or at international level, and fluency in English. Those who did not meet these criteria were excluded. Twelve experts were contacted by email and introduced to the study, its objectives and the involved researchers. Those who responded were recruited for this study, but two respondents were unable to attend the interview due to their busy schedule, leaving a final sample of 10.

FHCWs included clinic managers, PMTCT nurses and midwives in clinics randomly selected from the clinics under the Public Health Department of Cape Town or Western Cape province, South Africa. Their recruitment occurred alongside the recruitment of experts. Eligibility included the following: managers of the local facilities offering PMTCT services, nurses or midwives delivering PMTCT services or dealing with PMTCT related problems in the clinics. Those without experience in organising PMTCT services or who did not get involved in its delivery were excluded. A total of 15 FHCWs were contacted by introductory email for the study with a follow-up telephone. Only 10 were available and recruited for this study. Five who chose not to participate cited different reasons including conflicting agenda and urgent matters to attend to in their facilities.

#### Data collection

Participants completed the face-to-face interview at their workplace or any other suggested quiet setting. A self-administered questionnaire was used to collect sociodemographic information including age, sex, years of experience and institution or centre of affiliation before the interview. Interviews were conducted by a trained male researcher (JCM) under the supervision of a female experienced qualitative researcher (KM). The researcher (JCM) introducing himself as a doctoral student and briefly interacted with the participants about the study before commencing the interviews. Each interview lasted between 30 and 60 min, a timeframe in which the data saturation was reached. A semi-structured interview guide (Additional file 2) was developed by JCM and revised by CZ, and was used for both groups of respondents, experts and FHWCs. The development of the interview guide included a review of various PMTCT policies and implementation guidelines from both the World Health Organisation (WHO) and the South African Government, and questions were generated from these guidelines. Open-ended questions were used to explore participants experience of PMTCT service integration into PHC. The interview guide was modified over the course of the study, using an iterative process informed by the content of previous interviews. For example, questions regarding health information systems and PMTCT data and related statistics were added to understand how information and datasets are shared among various health professionals and institutions, and how monitoring and evaluation of PMTCT services were carried out. The interviewer used reflective probes to encourage respondents to clarify and expand on their statements. Interviews were ongoing until no new information was obtained and thematic saturation was achieved by the end of all scheduled interviews for both groups of study participants, experts and FHCWs. All participants were offered no compensation.

#### Data analysis

All interviews were audiorecorded and field notes were taken by JCM, and a verbatim transcription was conducted by a hired and trained assistant. Using an iterative approach to thematic analysis [51, 52], two investigators (JCM and CZ) independently read de-identified transcripts, identifying meaningful segments within the responses. All interviews were coded by JCM in collaboration with CG and CZ, and all discrepancies in the coding process were negotiated between these three investigators. The CAS framework was first applied in the process of identifying relevant categories and later in the interpretation of the themes that emerged from the transcripts. General themes and subthemes summarizing perspectives of the two groups of participants (experts and FHCWs) were discussed among members of the research team, and the representative quotes presented in this article were collectively selected. Atlas.ti software was used to assist data analysis and management. Characteristics of participants were analyzed by JCM using SPSS version 24.0.

#### Ethics approval

The study was approved by the Human Research Ethics Committee at the Faculty of Health Sciences, University of Cape Town (HREC REF: 946/2014) and the Centre de Recherche du Centre Hospitalier de l’Université de Montréal (2018–6690, 17.044 - ID). Written informed consent was obtained from all participants.

## Results

### Participant characteristics

Twenty participants were interviewed; 10 experts and 10 FHCWs. FHCWs included three PHC clinic managers and seven PHC clinic nurses. All recruited FHCWs were working in public local health facilities located in the disadvantaged communities of Cape Town suburbs including informal settlements. They normally serve a population overburdened by infectious like TB, HIV and others, but also chronic diseases including hypertension and diabetes. These FHCWs deal with enormous work pressure to offer comprehensive and quality care in these communities. The majority of interviewees in both groups were female (80%), with an age range of 29–61 years (mean age 45 years) (Table 2).

**Table 2:** Study participant characteristics

Participant characteristics	N (%)
Participant category	
Experts	10 (50)
FHCWs:	
Clinic managers	3 (15)
Nurses and midwives	7 (35)
Sex	
Female	16 (80)
Male	4 (20)
Age mean and SD:	
Experts	49.8
FHCWs	40.1
Overall mean (SD)	44.9 (8.2)

*SD* Standard Deviation

### Themes and categories

The key themes that emerged from all interviews were grouped into seven categories. These categories were then mapped onto three components of the adapted CAS framework, organised by the occurrence of events throughout PMTCT rollout during and after AIDS denialism. They were classified as: “system” (experiences in trials and PMTCT adoption during AIDS denialism era); “adaptive” (gradual move towards PMTCT acceptance and integration, increased awareness and community participation, commitment to PMTCT services integration, community health workers’ involvement, clinical training and retraining for nurses) and “complex” (persistent barriers to PMTCT integration). This classification with illustrative quotes is depicted in Table 3.

**Table 3:** Key findings based on proposed CAS components

CAS component and key findings	Illustrative quotes
**System**	
1. Experiences in PMTCT trials during and after AIDS denialism era	*“We had to get special permission to roll out the programme, and… when there was a court instruction to roll it out; but then they were still controlling the roll-out and we were all, like, pilot projects” –* Expert 2.
	*“I think over the years what has happened is the pressure on the ground that has resulted in people changing their perspective and focus around PMTCT”* – Expert 1.
	*“I’m just thinking back now, in general and for Cape Town in particular,…So basically, we received global funding, and in 2006, we appointed quite a number of PMTCT coordinators, as well as PMTCT registered professional nurses within the PHC setting…”* – Expert 4.
	*“It was only AZT and Nevirapine that was given at that time. Then as time goes by then we changed”* – FHCW 1.
	*“.....she started to run the PMTCT programme about 15 years ago, lots of patients were lost before that and statistics were quite high when it came to PCRs. They weren’t followed up appropriately. So, I think after the PMTCT programme started, there was somebody actually taking charge, invested in the patient’s wellbeing, and they were being followed up, and if any other issues arose besides PMTCT, it could also be managed more appropriately and be referred”* – FHCW 2.
	*“I believe there was a problem with a theft on medication. As a result now, the nurses that are doing ANC, they don’t dispense medication”* – FHCW 3.
**Adaptive**	
2. Commitment to PMTCT integration	*“And in some settings, actually referring the patient involved having them moving from the facility to another to obtain the care for other conditions; and you’ve got to think of integration in terms of, at least, the making available within the same facility of other services”* – Expert 6.
	*“…transferring clients to specialists may cause gaps and some women become missing, even for a short distance…, they disappear within 50 m to where you send them. They fall in hole in that walk and with this we can’t defragment the system”* – Expert 10.
	*“It’s a logical move for midwives and other health workers who are working in maternity, antenatal, and postnatal and delivery services to treat mothers who are infected with HIV in a holistic way”* – Expert 3.
	*“It’s quite good, because we try to integrate services…..So we do the PCRs when the mothers are here for immunisation, I mean, it can be sooner done at once”* – FHCW 3.
	*“You want your baby to be immunised here; then we don’t want you to go up and down, you must also be here and your baby, so that we can see both of you ….But the mother and the baby must be on the same facility”* FHCW 4.
	*“…because they see mother and child, then they also check which medication did the child get, or is the child a high risk infant or not? Does the mother still remain in care? All those check ups, PAP smears, they also check immunisations, deworming, nutrition,…everything possible”* – FHCW 5.
3. Gradual move towards PMTCT acceptance and integration in the clinics	*“…PMTCT figures almost synonymous with antenatal care, because the chances of being HIV infected are so high, relatively, that you almost don’t see one without the other”* – Expert 8.
	*“So most of our nurses, our midwives are now trained, so which means they’re offering integrated antenatal and antiretroviral treatment (PMTCT) and they’re also doing TB screening”* – Expert 4.
	*“Western Cape was the only province to start integration of PMTCT and other ANC services and others did the same later. Different stakeholders used to meet once per week to discuss the progress and issues and everyone came together to assess how it was managed”* – Expert 9.
	*“The community is more open-minded now of what is expected of them once they are pregnant…So, it was an eye-opener, also for the nurses, because and the community as well; because now they know, once they are pregnant, they are eager to know their status”* – FHCW 6.
	*“…within the facility, it’s now good that we realised that there is a need for that continuity of care…..when we started this project of Child Health”* – FHCW 1.
	*“I can say one in 5 years, we have only one child, one in 5 years that is positive. She didn’t come to the clinic when she was pregnant, and yet she was positive. You see, it can take one in 5 years for one child due to PMTCT; and we are not happy with that”* – FHCW 4.
4. Increased awareness and community participation	*“It has so many adverse consequences affecting key public health indicators, such as maternal and under five and infant mortality, that it became a priority health problem for the health services to solve”* – Expert 3.
	*“…bringing HIV care back to the community, now increasingly having what they call community-based club, and you are going to see for instance more room for those people, to deal with more urgent issues, because they are overcrowded”* – Expert 6.
	*“….community support also plays a key role in supporting moms throughout this whole cascade. Peer support is important”* – Expert 10.
	*“We have got programmes like MomConnect where they are enrolled and they get all the support, like where they are sent sms’s and stuff”* – FHCW 6.
	*“Mothers to Mothers services which is well supporting the mothers who are pregnant and those after delivery, so they work hand in hand with antenatal labour ward as well as Child Health for continuity of care”* FHCW 1.
	*“So, when we talk to them we get the social worker involved as well, then we will try and make a plan to see if there are not other family members because, I mean, any support, it doesn’t just have to be the partner, support in general is good”* – FHCW 2.
5. Community health workers’ involvement	*“CHWs are part of health care system to link clinics to the communities and enforce adherence among their other roles. They need to be trained”* – Expert 9.
	*“…if they were in place, in many communities, it would be relatively easy to send a message to those community health workers and teams”* – Expert 3.
	*“They (CHWs) can go to the houses and they can reinforce adherence…and the idea is that they go into that household and look at all the problems. They look at the teenagers, they counsel them about contraception, and look at the mother that’s struggling with breastfeeding and at the father that’s smoking”* – Expert 5.
	*“I will say they are helpful, because we use them as the bridge just to get to the communities, because they go the extra mile in this way that they go inside those houses; then they will go there and give talk…So we work hand in hand with them”* – FHCW 7.
	*“…but mostly our community health workers are working with TB and HIV,… Only if, for example, we need to recall our babies for immunisation…. then they will recall those mothers for us.* W*hen we are doing some bloods screening and there is an abnormality, they can’t get that person from the phone, and then somebody has to go and do a home visit*” – FHCW 3.
	*“After delivery, CHWs maybe they visit them (women) at home”* – FHCW 8.
6. Clinical training and retraining for nurses	*“We trained nurses and they are delivering ART and none believed that in those days, training changes everything….”* – Expert 10.
	*“…When everyone diagnosed with HIV was to be initiated to treatment and nurses had to be train on ART initiation. NIMART was created and trained them”* – Expert 9.
	*“We said that the NIMART nurse who’s got a dispensing licence can also dispense the medication in the MOU, to make sure that the mom gets more of an integrated service…”* – Expert 4.
	*“In South Africa, for special training, they have to do the NIMART training, and then they can dispense the drugs and look after the patients and the counselling…”* – Expert 5.
	*“We wanted all the midwives to be NIMART trained, so they would be able to initiate, so they don’t send the patients from pillar to post. Then they get tired and don’t start their medication because they must wait in other queues”* – FHCW 1.
	*“….just to send the staff to the trainings on PMTCT, on BANC….yes, I see who is running short, so that I send them for training through NIMART training programme”* – FHCW 4.
	*“I do BANC and most of the time… I don’t have the HIV course, so I must run around and asking those sisters there for my patients all the time; and if they would send me for HIV training that would be resolved”* – FHCW 8.
	*“…because, the staff have to be trained, and especially on the PMTCT guidelines when they change again, so, they change a lot and there should be uniformity”* – FHCW 3.
**Complex**	
7. Persistent barriers to PMTCT integration	*“If we have an electronic system that talks to each other and the patient has got a single number, we can track her…it’s not installed in all the computers and not everyone is trained how to use it...Other health problems like Hypertension is not integrated into the PMTCT services”* – Expert 5.
	*“I think that there was resistance to change. I remember there was a time, just before we introduced the same day initiation. When we spoke to nurses about it, they said it’s impossible, we can’t do it; and so there are attitudinal issues and they needed support and training and encouragement. So people don’t really want to change necessarily, but when it’s policy, you say, well we don’t have a choice”* – Expert 7.
	*“Some mothers book late, and so don’t benefit from the services available. Other mothers convert after they’ve been initially tested and they’re especially at high risk, so identifying and getting the mothers onto treatment is one of the big challenges; and then keeping mothers adherent is another challenge”* – Expert 3.
	*“We wanted all the midwives to be NIMART trained, so they would be able to initiate, so they don’t send the patients from pillar to post. Then they get tired and don’t start their medication because they must wait in other queues”* – FHCW 1.
	*“…for stigma purpose, because they know in our communities if you breastfeed then you are regarded as negative….So we have that challenge, breastfeeding for two weeks then stop, swap to formula feeding”* – FHCW 7.
	*“I believe there was a problem with a theft on medication. As a result now, the nurses that are doing ANC, they don’t dispense medication. So now those mothers, they queue twice”* – FHCW 3.
	*“Our waiting area is very congested. We don’t even separate them, you know, like kids as such and mothers…, and also there’s poor ventilation in there,… and then they come here and actually, those kids may leave this place more sicker” –* FHCW 3.

The following section discusses the results and demonstrates the sequence and complexity of integrating PMTCT services within PHC in South Africa.

#### Experiences in PMTCT trials and its adoption during AIDS denialism era

From the perspectives of HIV experts who were involved in the initial PMTCT trials, during the era of AIDs denialism, doctors and the other health care professionals, community groups and activists representing people living with HIV were the first to push for access to HIV treatment. These early actors in the campaign for treatment for mothers and children had tremendous and diverse experiences of how PMTCT was rolled-out during and after AIDS denialism. Several experts interviewed had participated in the design and implementation of PMTCT in local pilot sites and at national level. FHCWs interviewed had not been involved in the early stage of PMTCT rollout.

Experts, who to some extent played a key role in advocacy for HIV treatment or against AIDS denialism, recalled the introduction of PMTCT. The majority discussed how unsupportive the Department of Health (DoH) was, and the difficulty in obtaining approvals at local clinics. Six experts who were involved in the initial PMTCT trials in South Africa described how the PMTCT intervention was vertically rolled-out in selected pilot sites prior to and following the constitutional court ruling in August 2002.

*“In South Africa, when we started the PMTCT Programme, it was really a programme implemented in 18 pilot sites, and then based on a court order, we had to scale it up nationally, and at that time, it was very much a vertical programme*” – Expert 1*.*

Though campaigns to prevent MTCT had legal support, it took time for the DoH and all health care providers to fully cooperate with the teams that were delivering HIV interventions. Researchers and activists who were involved in the scaling-up of PMTCT interventions recounted how they personally sought support from health care providers, clinic committees and from pregnant women involved. Bringing together these different groups of stakeholders was essential in gaining support for implementing complex interventions such as PMTCT.

*“We would first meet with the clinic… Every clinic had a clinic board made up of lay people and community leaders in that district; and so, the first strategy was to get those people to be on your side”* – Expert 2.

In contrast, the majority of the FHCWs knew very little about how PMTCT services were first implemented into PHC in South Africa. Through training and positive outcomes that followed PMTCT rollout in their facilities, FHCWs have learnt the importance of PMTCT within PHC package but most (*n* = 7) were not able to comment on the history of its integration. One clinic manager assumed that PMTCT had been integrated from the beginning.

*“It’s always been integrated, so I don’t want to say something that I don’t have experience of. I don’t want to talk based on assumptions”* – FHCW 1.

Another clinic manager who worked at the pilot site was unable to recall what happened when PMTCT services were offered for the first time in her facility. Like others with the same experience, stories were short, imprecise and with few details on how PMTCT services were initiated and later integrated into PHC.

*“We started by piloting it in 1999. We were the pilot site. I must try to think now where I was working in that time of the PMTCT; because I remember these babies, they were HIV affected. You know, it was that way.…. We didn’t even have registers, at that time, because we didn’t even know how big the problem was at the time”* – FHCW 2.

#### Gradual move towards PMTCT acceptance and integration in the clinics

Most experts involved in this study reported that they were among the teams of researchers and activists that were pioneering this intervention and worked to mobilise buy-in and support for PMTCT at an individual and organisational levels. They opposed the government’s stand, and worked with clinic boards and community leaders in every district for the acceptance and initiation of PMTCT, as illustrated in the following quote:

*“It was in the beginning of AIDS denialism, and so the way we entered into the clinic, was we would first meet with the clinic/hospital, and if the clinic/hospital says, we sanction this, it’s very hard for the clinic staff not to allow you into the hospital”* – Expert 2.

According to the experts who witnessed the whole evolution of PMTCT scale-up in South Africa, despite the Constitutional Court ruling in favour of PMTCT rollout, research teams, activists and NGOs continued to face challenges in efforts to implement the intervention in most of the provinces. Those championing the rollout encountered resistance from FHCWs who worried about HIV transmission to themselves and that the intervention would add to already heavy workloads. Meanwhile, HIV-related health care needs were increasing in the country, especially among pregnant women attending clinics for antenatal care, which put healthcare workers under increasing pressure to respond. Health care research teams were also inspired by the civic-minded attitude of those women who willingly participated in early PMTCT trials to support efforts to rollout services to more women. These women were hailed as champions.

*“… When there was a court instruction to roll it [PMTCT] out; we were all, like, pilot projects. We were doing rollouts, but it was always very controlled…. it was a placebo-controlled study, and the women said to us, “we don’t mind being on placebo, as long as you promise us that if this drug works, you will roll it out, and you will give it to all other [women]… So, we will sacrifice ourselves for the future women”. So we promised the women that if it worked, we would do whatever it took to make sure that other women in their situation [received the intervention]…”* – Expert 2.

All FHCWs interviewed spoke unreservedly about the benefits of the PMTCT programme, reporting that they had progressively embraced new initiatives and expressed their full support for PMTCT integration into PHC services. They saw how PMTCT integration at a facility level helped to deliver services to community, and how this had become crucial for the successful delivery of other services for maternal and child health. A researcher who participated in PMTCT rollout from the very beginning and a manager working at the PHC level discussed how integration of PMTCT services in local health facilities benefited other services.

*“..Mother to child transmission is such a big problem. It has so many adverse consequences affecting key public health indicators, such as maternal and under five and infant mortality, that it became a priority health problem for the health services to solve. So the natural home for PMTCT is within the main stream services”* – Expert 6.

*“With the integration, it’s the best thing that ever happened, and also where we are now, compared to ten years ago, it’s like a big improvement; from the infection perspective-wise, of cross-infection and stuff, from mother to child”* – FHCW 1.

Despite strong support from women and FHCWs, the rollout faced a number of challenges. Addressing HIV related stigma and increasing programme acceptance required support from community-based NGOs. NGOs included those with a special focus on treatment like Médecins Sans Frontières (MSF) and those with activism and advocacy aims like TAC. Experts and FHCWs who worked with NGOs on a daily basis in their local facilities praised them as key players in PMTCT intervention acceptability, in retaining women, or in boosting support from families of women attending PMTCT with their babies after delivery. Four FHCWs and three experts discussed how NGOs have helped from the very beginning - from awareness campaigns, assisting in the training of lay health workers and equipping clinics, as well as providing support and follow-up with women in their communities. NGOs using community health workers (CHWs) and lay counselors continue to play a vital role in working with national health department to reinforce governmental initiatives dealing with MTCT and increasing women retention.

*“…So it has improved a lot based on other NPOs* [*non-profit organisational staff*] *that assist us if we have a problem, when we can’t find a patient, then mothers trace them, or the NPOs trace them*” – FHCW 3.

#### Commitment to PMTCT services integration

All participants discussed knowledge of health care services integration and their experience in the process of integrating PMTCT cascades in PHC. Experts considered PMTCT integration as the ‘right process’ to bring together clinical activities. All experts believed that PMTCT fitted well within other maternal and child health (MCH) services and felt that it had been smoothly integrated into other available services in the clinics such as tuberculosis treatment, sexually transmitted infections and mental health services.

*“It’s linking better with the MCH programmes… On the ground, I think, at facility level, there always is integration”* – Expert 1.

FHCWs who had been involved in service delivery at the clinics understood how challenging it had been to integrate various services in their facilities. However, they recognised the importance of PMTCT integration in daily service provision at the clinics. In striving to deliver a package of comprehensive services, PMTCT became the core component among the services offered at their local facilities. For example, one of the FHCWs shared how she worked to ensure all services were integrated for the convenience of women.

*“So, what I did, I tried to integrate everything, and to make sure that whoever is working here is going to give a one-stop-shop… They [the woman] are going to get all the services they are supposed to get, instead of going there and there and there”* – FHCW 1.

#### Increased awareness and community participation

In addition to combined efforts from both national and provincial health systems, local authorities and NGOs to increase PMTCT awareness, community involvement was also essential to increasing uptake and sustainability of the programme. PMTCT mobilisation and integration campaigns have therefore included community-members to share first-hand messages and experiences to peers, relatives and neighbours and ensure the intervention is widely known and accepted. The following two groups were, based on our interviews, identified to have carried out important advocacy and treatment messages about PMTCT and boosted its successful implementation: mothers-to-mothers, and male partners of women who accessed PMTCT.

### Mother-to-mother services

Barriers to accessing services, treatment initiation and adherence to it, were acknowledged by both experts and FHCWs. Mother-to-mother (M2M) was perceived as the core peer-support organisation to offer services that helped to address these challenges. First initiated and registered as an NGO partnering with other governmental, multilateral and individual organisations, M2M started in South Africa employing trained mothers living with HIV to provide psychosocial support to pregnant women and mothers of babies diagnosed with HIV, to promote retention and to encourage disclosure [53]. Strong support for mother-to-mother’ initiatives was expressed by both experts and FHCWs:

*“Mother-to-mother initiatives have been fantastic for making sure moms come back and take the medication…community support also plays a key role in supporting moms throughout this whole cascade. Peer support is important”* – Expert 3.

*“Mothers to Mothers services which are supporting the HIV mothers who are pregnant and those after delivery, work hand in hand with antenatal labour ward as well as Child Health for continuity of care”* – FHCW 3.

Both groups of participants also discussed how the M2M peer support group meetings were forums in which women discussed appropriate solutions in responding to HIV infection in their communities, as well as issues they experience, such as difficulties with treatment adherence, social support and poverty. M2M also provides an opportunity for health education and counseling to address any concerns and increase understanding of HIV transmission, MTCT and basic health preventive health practices.

### Male involvement in PMTCT

Active involvement of males in supporting pregnant women living with HIV was regarded as important by both experts and FHCW. However, experts described how antenatal and postnatal health services in the country are ‘womanised’ - a situation that did not foster male involvement in services. Both experts and FHCWs emphasized the need for more efforts to bring in partners to services to improve PMTCT.

*“Male integration is something else we didn’t do well and it needs to be improved a bit…and our health services are female oriented and male staff and partners of our women clients are to (should) be more involved”* – Expert 3.

FHCWs found male support and collaboration difficult to gain:

*“Other contributions were involving other… allowing people to be involved from outside, especially the men, because our African men don’t go to antenatal….to promote the partners to come with”* – FHCW 3.

#### Community health workers’ involvement

While there is a long history of CHW involvement in PHC, the advent of HIV and of treatment for HIV and tuberculosis in South Africa led to a major increase in the numbers and visibility of CHWs such as lay counsellors. Many were employed by NGOs and funded by external donors but working in close collaboration with public health facilities [54]. For experts, CHWs were a cornerstone for linking communities and health systems, addressing stigma and discrimination at family and community level, and for supporting medication adherence. They emphasised the need to establish or enhance community-based services (CBS) with CHWs engagement and full collaboration with health systems.

*“They (CHWs) are more available than other health workers in the clinics.…and if you go back to the HIV story again, I mean, using CHWs is all about bringing HIV care back to the community as they are in charge of what they call community-based club,…. and this gives more room for those nurses and others in clinics to deal with more urgent issues, because they are overcrowded now. It (the club) gives them that repeated contact and a lot of those redundant tasks can obviously be shifted nicely to a community health worker who is trained and accessible, and then that eases the pressure on the clinic”* – Expert 4.

Experts valued the contributions of CHWs, especially in responding to HIV and in facilitating PMTCT implementation. Increased training and appropriate payments for CHWs were suggested by experts, to ensure that they are motivated and are able to support communities in partnership with local health facilities. Experts experience was that, despite intentions that NGOs provide adequate training, supervision and payment, this was done so inconsistently.

*“That systems of peers and CHWs, they need to be properly trained and paid, that’s my opinion…When they are trained and offer their services, they have to be paid”* – Expert 3.

FHCWs also recognised the importance of CHWs at different fronts to link local health facilities and the communities. Three FHCWs stated they had reservations about the involvement of CHWs but acknowledged that they are useful in ensuring follow-up of women and continuous PCR testing and immunisation for babies. Some CHWs were thought not to have the required education to support community members. This meant that continuous training and supervision by the designated clinic nurse or midwife was required to ensure that the right information and quality services were delivered by CHWs as suggested by one clinic manager:

*“….because I need first somebody to train them, or maybe somebody senior to them to listen to what they are saying, because people they start to ask questions. We also see that as an option and then some of them don’t even have grade 12, so, those kinds of things. So, we try to control it…”* – FHCW 2.

However, most FHCWs described collaboration with CHWs as critical to the success of the PMTCT programme and facilitating its reach into the surrounding communities. CHWs supported the delivery of health education for a range of health issues, located mothers for appointments if they could not be reached via phone and organised HIV/AIDS support groups at antenatal clinics.

*“I will say they are helpful, because we use them as the bridge just to get to the communities, because they go the extra mile in this way that they go inside those houses; then they will go there and give talk…So we work hand in hand with them”* – FHCW 5*.*

#### Clinical training and retraining for nurses

Given the challenge of doctors providing treatment to a large number of people living with HIV, South Africa has adopted a decentralised approach, allowing other health professionals to prescribe treatment. The Nurse Initiated and Managed Anti-Retroviral Therapy (NIMART) training programme was established as an effective task-shifting strategy, following the conclusion of the Streamlining Tasks and Roles to Expand Treatment and Care for HIV (STRETCH) study in South Africa in 2010 by Fairall L. et al. [55]. Its role was to train nurses to not only test HIV, but prescribe ART support to pregnant women living with HIV. It was regarded as successful by both experts and FHCWs but they described several challenges in the roll-out of this programme. The shortage of NIMART trained nurses and midwives was thought to result in high workload for those trained and was linked to long waiting hours in the queues and poor-quality services.

*“Well, I think in the facilities where you’ve got enough NIMART trained nurses, there isn’t a problem…”* – Expert 5.

*“We wanted all the midwives to be NIMART trained, so they would be able to initiate, so they don’t send the patients from pillar to post. Then they get tired and don’t start their medication because they must wait in other queues”* – FHCW 3.

*“Because I do BANC [basic antenatal care] and most of the time… I don’t have the HIV course, so I must run around and asking those sisters there for my patients all the time; if they would send me for HIV, then it’s going to be okay…” –* FHCW 6.

Frequent changes in PMTCT and ART guidelines also presented challenges in ensuring all staff members understood and were implementing updated guidelines.

*“I think it is still challenging, particularly when we have to implement new guidelines, because we’ve got to go through a whole process of training and retraining. And the trouble is with the whole HIV and PMTCT field, is evidence evolves and guidelines change rapidly or consecutively in close succession, so we need to keep retraining groups of people” –* Expert 1.

In particular, several experts and FHCWs mentioned changes in the guidelines about breastfeeding. While HIV mothers were previously recommended to formula feed, mothers who have a supressed viral load are now being asked to breastfeed consistently. This has resulted in confusion for both staff members and mothers.

*“The other challenge was consistent messaging, because you know, different people were saying different things, and there was a lot of confusion about infant feeding”* – Expert 1.

#### Persistent barriers to PMTCT integration

Like other complex health programmes, PMTCT rollout has faced challenges in South Africa (Table 4). Experts mentioned attitudinal issues from facility-based health care workers and lack of accountability and bureaucracy at all levels of management in the health system. They also discussed how poor communication and inconsistencies in the fidelity to the clinical algorithm and practice guidelines reduced the overall quality of care, in some facilities.

*“I think it’s due to a lack of accountability and a lack of good management at all levels…And currently, there’s no great communication between primary level and hospital level generally, referral both ways”* – Expert 6.

Experts suggested that a single health information system has the potential to improve efficiency in patient identification, follow up and reporting and take its integration to a new stage.

*“So, we know patients are nomadic… we need linked electronic patient records, and the problem is for HIV care for example in this city, they have three different systems”* – Expert 5.

FHCWs also expressed frustration regarding the lack of communication between clinics and their referral hospitals and the lack of a national-wide system of unique identifiers for all patients.

*“If we transfer them, they must take over everything, but the problem is, if the patient went to a hospital, even if she went to our Khayelitsha District hospital, if they give them medication there, it doesn’t appear, because we don’t know where they captured them. So, they appear to us as defaulters, up until we must phone them”* – FHCW 8.

FHCWs discussed how their heavy workload, inadequate training, staff shortages and high staff turnover affect the quality and outcomes of service delivery in their health settings.

*“It was not all smooth sailing, due to staff turnover…..I had, like almost, six, seven nurses who were basic antenatal care [BANC] trained, who can do all for the pregnant mother… from the time that their patient is pregnant until the baby is born, and still look after the baby, but then with the staff turnover, they left and it was tough, terrible! I’ve got only two people who are BANC trained, and these people, it’s not like the only thing that they must do. They were doing other things as well, and not only focussing on this* [PMTCT]” – FHCW 1.

**Table 4:** Persistent barriers to PMTCT integration

According to experts	Common to both experts and FHCWs	According to FHCWs
•Bureaucratic slowness •Lack of managerial accountability •Poor quality of care including suboptimal fidelity to algorithms •Poor training of staff •Attitudinal issues among health care workers in the facilities •Discrimination, stigma •Lack of national health information systems and issues relating to electronic records	• Loss to follow-up • Socio-economic issues for women • Women migration • Lack of unique patient identifiers • Resistance to change	•Small working spaces and other infrastructural related challenges like poor ventilation, congestion, among others •Shortage of personnel and work overload •High staff turnover •Guideline changes •Too much paperwork for managers

## Discussion

Political support of AIDS denialism at national level resulted in the deaths of many mothers and children, and delayed South Africa’s adoption of PMTCT until 2002 [30]. Since then, South Africa has been leading the development of PMTCT integrated into PHC in order to address broader maternal and child health care. This study adds to the limited literature on what has enabled, and challenged, the integration of PMTCT from the perspectives of both FHCWs and experts.

The interviews highlighted that commitment and ground level collaborations between different stakeholders, including policy, research and activist groups, FHCWs and women from the broader community were essential to navigating around the political climate to ensure the integration of PMTCT services. Considering health system and community elements and their interactions is key to the success of interventions or programmes and should be looked at in terms of degree of interrelatedness and actors from health system structures, their understanding and functions [56] as demonstrated in the study conceptual framework. While the rollout of PMTCT was met with resistance early on from FHCWs, they slowly joined the efforts to integrate, agreeing with experts on the importance of PMTCT integration within existing services, especially those related to MCH. As PMTCT care was incorporated into well-equipped facilities where trained doctors and nurses were available, NIMART training contributed to a decentralised approach with a special focus on women and children from general HIV care. As the task-shifting model aimed to overcome issues of low doctor/patient ratio, PMTCT integration was seen as relatively successful by all participants. However, consistent with what happens in other countries, FHCWs argued that beyond focused training on PMTCT algorithms, workload and infrastructure constraints have impeded work towards full integration and eliminating MTCT in South Africa [57–59]. Apart from NIMART that has not been offered to all nurses due to many not reaching the qualification threshold, PMTCT protocols have continued to evolve and require compulsory both off-and on the-job training – which not all FHCWs are able to attend, resulting in PMTCT integration in some local facilities being limited.

The need to involve other groups outside of the health system from the outset for training, treatment, advocacy and community mobilisation (such as MSF, TAC, M2M or other well-equipped NGOs) to reinforce PMTCT services integration was acknowledged by all study participants. Such collaborative initiatives were required from the very beginning to manage the health needs of pregnant women and their babies [60]. NGOs and partnering local community groups supplemented overwhelmed FHCWs to implement various programmes at local health facilities. Despite the criticism of competition for funding rather than cooperation with national, provincial and district health departments [61], this experience of collaboration was positively regarded by all study participants. Mother to mother services were highlighted by both experts and FHCWs as a way to reduce MTCT and strengthen community-based support. Participants credit M2M efforts and services with establishing links between PMTCT mothers and their local PHC facilities, and maintaining them through the many elements of the PMTCT cascade [62]. A study conducted in Zimbabwe by Shroufi, A. et al. [53] has similarly found that M2M increases retention in PMTCT, owing to their community-based approach. The three M2M core objectives of 1) reducing the number of babies infected with HIV during or after delivery, 2) promoting the health of pregnant women and new mothers and increasing their opportunities to access and effectively use health and life-sustaining ART, and 3) supporting disclosure and fighting stigma and discrimination [63] have been generally achieved in the SA context [63–66]. Participants in this study explained how and why the CHWs are increasingly accepted into PHC. CHWs have emerged as key players in response to HIV/AIDS, tuberculosis and the Ebola virus, among other health problems – particularly in overwhelmed health systems like in South Africa [67–70]. In case of PMTCT services, the CHWs’ role has evolved to improve health for both mothers and their infants, as well as for other family members in the households in different areas of health priority such as disease prevention, hygiene, nutrition, family planning, social support and domestic abuse [71–75]. Even though critical, investment in CHWs in South Africa has to date been through NGOs partnerships and have not yet been formally institutionalised nor thoroughly documented despite being considered as a key element of PHC re-engineering strategy, launched almost a decade ago [70, 76, 77]. This situation does not motivate nor facilitate the development of CHWs, who are mostly from a low social-economic background, in their commitment to advance health care services in communities and at the local facilities. Continuous in-service training, adequate supervision, proper financial incentives and other benefits could bridge the gaps in CHW’s knowledge and ability to work closely with PHCs – issues that currently can raise ethical questions in relation to quality of care [78].

Both experts and FHCWs were enthusiastic about the positive results already achieved by PMTCT but were dismayed by HIV stigma and discrimination, small and insufficient spaces and other infrastructure related challenges in the facilities. They did, however, diverge on experiences regarding other health system challenges that impede the full integration of PMTCT within PHC and consequently the virtual elimination of MTCT in South Africa. Experts insisted on bureaucratic slowness and lack of managerial accountability, poor quality of care, including suboptimal fidelity to algorithms and remnant attitudinal issues in the facilities as obstacles. FHCWs expressed concerns with work overload, high staff turnover, loss to follow-up of women, shortage and poor training of staff. These challenges, among others, are well documented to have historically affected the quality of health care that is a constitutional obligation in South Africa [79, 80] and have indeed slowed down the progress with regard to PMTCT integration and virtual MTCT elimination in the country [29, 81]. Participants from both groups suggested approaches and initiatives to address these complex common issues faced by health systems in South Africa and in other LMICs as illustrated in this study conceptual framework. These measures include investment in infrastructures, adequate training of workforce in various capabilities, use of electronic unique patient identifiers and health information technology, social assistance to facilitate transportation, as well as increased investment in the fight against hunger and addressing a number of social factors contributing to HIV-related stigma and discrimination in which influence non-disclosure and low adherence rates [82–86]. This would be a tremendous step towards fully integrated PMTCT especially in the introduction of WHO recommended option B+ adopted in South Africa, in which all positive tested women are initiated to ART regardless of their CD4 cell count [82, 83]. Once achieved, full integration of PMTCT would serve as a PHC model to adequately deal with multimorbidity epidemics among ageing populations, including women living with HIV [87–89]. With more investment from stakeholders and consistent changes in bureaucracy and greater accountability from National Department of Health, PMTCT integration within other basic health services might be revamped and more fully reported on in South Africa [30, 90].

### Strengths and limitations of the study

#### Strengths

This study recaptured the history of PMTCT implementation at local PHC facilities and documented lost lessons or failures, as well as successes, that could be relevant for the integration of other interventions into routine primary care. It also included both experts and FHCWs who had different perceptions. Participants also ranged in their years of involvement in PMTCT implementation, and included those who had been involved in pre-2008 PMTCT services.

#### Limitations

Despite these strengths, this study also had limitations. Social desirability may have influenced responses and additional challenges may not be captured. Experts were from different areas, including policy, research, advocacy among others and this may have impacted their shared perspectives and experiences. The Western Cape and Cape Town have the best health systems and had hosted the first PMTCT pilot sites in the country - therefore, the practices and experiences of FHCWs working in this region may vary substantially from those of other provinces in South Africa. This study used a small sample size, and may not be representative of the experiences of experts and FHCWs across South Africa.

## Conclusion

While the integration of PMTCT into PHC has been hailed as a success, this research identified ongoing challenges in the integration process for HIV/PMTCT from both the perspectives of experts and FHCWs. Existing issues in bureaucracy and accountability presented barriers to full integration of PMTCT. For FHCWs, concerns of heavy workload and infrastructure constraints, and ongoing issues with training and high staff turnover created challenges in the care of both mother and child. South Africa has prioritised eMTCT – addressing such challenges in the integration of PMTCT into PHC will enable South Africa to achieve this.

## Supplementary information

**Additional file 1.** COREQ checklist.

**Additional file 2.** Interview guide for experts and FHCWs.

## Data Availability

The datasets analysed during the current study are not publicly available to preserve participant anonymity.

## Abbreviations

AIDS: Acquired Immunodeficiency Syndrome
ART: Anti-Retroviral Therapy
AZT: Azidothymidine or Zidovudine
CDC: Centers for Disease Control
COREQ: COnsolidated criteria for REporting Qualitative research
FHCWs: Frontline Health Care Workers
GFATM: Global Fund to fight against AIDS, TB and Malaria
HAART: Highly Active Anti-Retroviral Therapy
HIV: Human Immunodeficiency Virus
IQR: Inter Quartile Range
LMICs: Low- and Middle-Income Countries
MCH: Maternal and Child Health
MSF: Médecins Sans Frontières
MTCT: Mother-To-Child Transmission
M2M: Mother-to-mother
NGOs: Non-Governmental organisations
NIMART: Nurse Initiated and Managed Anti-Retroviral Therapy
PCR: Protein-Chain Reaction
PEPFAR: President’s Emergency Plan for AIDS Relief
PHC: Primary Health Care
PMTCT: Prevention of Mother-To-Child Transmission
SD: Standard Deviation
SSA: Sub-Saharan Africa
TAC: Treatment Action Campaign
USAID: United States Agency for International Development
WHO: World Health Organisation
