# Supplementary material for: Integrating the prevention of mother-to-child transmission of HIV into primary healthcare services after AIDS denialism in South Africa: perspectives of experts and health care workers - a qualitative study
Source: BMC Health Serv Res. 2020 Jun 26;20:582. doi: 10.1186/s12913-020-05381-5 (PMC7318762; doi:10.1186/s12913-020-05381-5)
Supplement: Supplementary file 2 — Additional file 2. Interview guide for experts and FHCWs. [file 12913_2020_5381_MOESM2_ESM.docx]

**Integrating the prevention of mother-to-child transmission of HIV into primary healthcare services after AIDS denialism in South Africa: Perspectives of experts and health care workers - A qualitative study**

**Interview guide with experts and FHCWs**

1. **Introduction**

Thank you for agreeing to be interviewed today. We appreciate your time. The reason why we have asked you to come today is because we want to learn out more about your experiences with the PMTCT programme and the care received by women with gestational diabetes at the clinic during ANC and post-partum.

There are no right or wrong answers to our questions. Different opinions and experiences are valuable to us and we hope you feel comfortable enough to be honest with us. If you have any negative comments or feedback you would like to give us, that is fine.

The information you share with us today is completely confidential. Your responses will not have any identifying information. Your responses will only be shared with members of the study team and will be compiled with other responses from various participants. We would like to tape our discussion because I cannot write down everything you say, but this will also remain confidential.

Before we start, we need to ask you to give us your consent. This means that this discussion is voluntary and you are free to withdraw at any time without having to give a reason.

Do you have any questions about the interview? Is there anything that is worrying you?

Do you have any *other* questions before we start the interview?

1. **Demographic information**

Names:

Age:

Sex:

Institution:

Position:

Years of experience:

Contact details:

1. **Interview guide**
2. We are interested in understanding how PMTCT has moved from being a vertical program, to one that is integrated into PHC. Can you share your perspectives of the key points, successes, and challenges with the integration history or process in South Africa?
3. We are hoping to learn from the PMTCT integration experience in order to help support integrated care for other health problems, including diabetes, hypertension, and others into PHC. Can you share your perspectives on whether and how other health problems or issues are managed or integrated in the guidelines and into the PMTCT services?
4. We would very much appreciate to hear your perspectives on how these processes are actually playing out on the ground in individual facilities and communities. From your engagement with the clinics, with the clinic manager (s), with the nurses/midwives or other health care providers, please can you describe your views of:

- How the clinic(s) are functioning in terms of PMTCT integration?
- Which have integrated PMTCT, at which level (extent) and why?
- How is postnatal care managed?
- What is/are the role of clinic manager (s)?
- What the role health care providers (Nurses and CHWs) are playing?
- What are the challenges and successes for integrating other health problems such as diabetes in post-partum for women under PMTCT programs? Probe: Whether the management of the clinics in jurisdictions are changing to integrate the care of these diseases, whether the GDM is specifically managed in PMTCT program and whether there is a planned post-partum follow up for women with subsequent GDM.

1. Finally, is there anyone in particular you think we should talk to next or any important documents or guidelines that we should consult?
